# X-ray fluorescence analysis of selected micronutrients in ten African indigenous leafy vegetables cultivated in Nairobi, Kenya

**DOI:** 10.11604/pamj.2019.33.296.19501

**Published:** 2019-08-13

**Authors:** Alix Dehayem-Kamadjeu, Justus Okonda

**Affiliations:** 1 Department of Physics, University of Nairobi, P.O. Box 30197-00100 Nairobi, Kenya

**Keywords:** EDXRF, spectroscopy, micronutrient, African indigenous leafy vegetables, daily value, EDXRF, spectroscopy, micronutrient, African indigenous leafy vegetables, daily value

## Abstract

**Introduction:**

There is a growing interest on vital roles of micronutrients in the maintenance of good health and enhancement of the immune system for prevention of diseases.

**Methods:**

Energy dispersive X-Ray fluorescence (EDXRF) spectrometer was used for qualitative and quantitative analysis of selected micronutrients Zinc (Zn), Iron (Fe), Magnesium (Mg), Calcium (Ca) and Potassium (K) in ten African indigenous leafy vegetables (AILVs) (Corchorus olitorius, Cucurbita moschata, Amaranthus blitum, Brassica carinata , Cleome gynandra, Solanum scabrum , Crotalaria ochroleuca, Urticadioica, Manihot esculenta, Vigna unguiculata) which are cultivated, marketed and consumed in Kenya and across East and West Africa.

**Results:**

In this study, the highest levels of Calcium, Zinc and Iron were found in Urticadioica leaves (75.0 mg/g), Manihot esculenta leaves (0.1 mg/g) and Cucurbita moschata leaves (1.0 mg/g). Amaranthus blitum leaves exhibited the highest content of Magnesium (9.5 mg/g) and Potassium (36.5 mg/g) respectively. This study demonstrated that based on weight and bioavailabilty, 10 g of Cucurbita moschata (dried weight) provides between 57% to 136% daily value of iron for children age between 7 and 10 years old and can provide up to 41%, 81% and 27% daily value of iron for female aged 18+, lactating females aged 18+ and males aged 18+ respectively. A 10 g portion of Urticadioica leaves (dried weight) will provide 75% or 58% of the daily value of calcium based on the North American or western European recommendation respectively while the same amount of Amaranthus leaves provides between 37% to 50 % of daily value of magnesium for adults of nineteen years and older based on their weight. The daily value of zinc despite its dependency with age, weight and Zinc bioavailability can be supplied by 10 g of Manihot esculenta leaves (dried weight) at a percentage ranged from 8% to 39%. Based on the 3510 mg daily recommendation, 10 g of Amaranthus, Brassica carinata, Cleome gynandra and Cucurbita moschata (dried weight) will provide 10.4%, 10.0%, 9.8% and 9.3% daily value for potassium respectively.

**Conclusion:**

The research findings are scientific evidences of the nutritional contribution of African indigenous leafy vegetables.

## Introduction

African indigenous leafy vegetables (AILVs) are part of the African indigenous vegetables (AIVs) whose natural habitat originated in Africa [1-3] as opposed to the traditional African leafy vegetables that were introduced over a century ago and due to long use, have become part of the food culture in the continent. Both African indigenous leafy vegetables and traditional African leafy vegetables have long been important components in African diets. They are indispensable ingredients of soups or sauces that accompany carbohydrate staples [4]. In the Plant Resources of Tropical Africa (PROTA), 6,376 plants are reported as African indigenous plants with information available on cultivation practices for 280 African indigenous leafy vegetables [5]. Even though a few studies have assessed AILVs' consumption [1,6,7], their share in a diet at the household level has not been adequately evaluated in Africa. Consumption of different varieties is influenced by the cultural backgrounds, hence some varieties are only found in certain communities [8]. It is interesting to recall that, our ancestors in Africa lived well for many years as they mainly relied on traditional foods that included the African indigenous leafy vegetables. Scientific study results show that such a population that relies on traditional food is less likely to suffer from cancer and other ailments [9]. Unfortunately changes in dietary patterns and food systems have led to increasing consumption of highly processed foods in many countries in Africa. Readily available and accessible, these products are often high in fat, sugar and salt and signal a shift away from traditional diets [10]. These changes have affected the consumption of African indigenous leafy vegetables as they are stigmatized and often associated with poor rural lifestyles and low status. There is a dire need currently to strategically reposition indigenous vegetables in the horticultural sector so that their potential can be fully exploited for food nutrition and income generation [6]. Scientific evidences of the nutritional contribution of AILVs are paramount for their integration into the global fruit and vegetable initiative for improved health. This paper presents the detection and quantification of selected micronutrients (Zn, Fe, Mg, Ca, K) in ten African indigenous leafy vegetables (Corchorus olitorius, Cucurbita moschata, Amaranthus blitum, Brassica carinata, Cleome gynandra, Solanum scabrum, Crotalaria ochroleuca, Urticadioica, Manihot esculenta, Vigna unguiculata) cultivated, marketed and consumed in Kenya and across East and Central Africa [3,7,11] using Energy Dispersive X-Ray Fluorescence (EDXRF) spectrometer.

## Methods

The vegetables were sampled in a peri-urban farm 20 km away from the city center of Nairobi and met the following criteria: the leaves are the primary edible parts, they were under cultivation but can also be gathered wild and are used by several ethnic groups. Table 1 present the different African indigenous leafy vegetables selected for the study. The leafy part of each type of the vegetables was washed with distilled water to remove soil and other earthy materials before being damped on a tissue to remove excess water and dried in an oven for 24h at a temperature of 90^0^ Celsius. Hand gloves were used during sample collection and preparation to minimize contamination. Dried vegetables were grinded and sieved using a standard laboratory sieve of 150 microns. For each vegetable, pellets were made in triplicate by mixing 2.4g of powder with 0.3g of starch as a binder. The mixture was milled in a pestle using a mortar for 10 min to maximize homogeneity and pressed into pellets of 25 mm to give reproducible irradiation and counting geometry. The pellets were then analyzed using Energy Dispersive X-ray Fluorescence Spectrometer (EDXRFS). EDXRFS is a multi-elemental technique for simultaneous analysis of samples (e.g biological samples) based on measurement of characteristic X-ray intensities emitted by the elements in the sample [12] following excitation with high energy X-ray beams. The experimental measurements were conducted using the Rigaku NEX CG Energy Dispersive X-Ray Spectrometer equipped with Rh X-ray tube source with Pd anode target, 50 W maximum power, 50 kV maximum voltage. NEX CG is powered by a new qualitative and quantitative analytical software, RPF-SQX, that features Rigaku Profile Fitting (RPF) technology. The software allows semi-quantitative analysis of micronutrients without standards and rigorous quantitative analysis with standards [13]. Optimized sensitivity, high peak/background ratio spectra was achieved using up to five (Mo, Al, Cu, Si and RX9) secondary targets with polarized optics instead of conventional direct excitation optics in a wide energy range. The characteristic radiation emitted by the elements was obtained by irradiating the sample for 50 s. The fluorescent X-rays were collected by a silicon drift detector (SDD) affording extremely high count rate capability with excellent spectral resolution of 140 eV for *MnK*α at 5.9 keV and data acquisition dead time of less than 25%. The concentrations of individual elements were determined by using fundamental parameter method in built in RPF-SQX analysis software in which matrix effects were counted for. The precision of the instrumental method and analytical procedures used was checked by triplication of the samples, running each sample four times. The method was validated using NIST Bowen's Kale as certified reference material (Ken/0/003/S.636 F, 1983) prepared under the same conditions and analyzed under the same irradiation conditions with the samples. The relative standard deviations (RSDs) were then calculated to determine the precision.

**Table 1:** List of leafy vegetables showing their sample codes, common, Kiswahili and botanical names

Common name	Kiswahili name	Botanical name	Sample Code
African kale	Kanzira or Kandhira	Brassica carinata	Kan
Amaranths leaf	Mchicha	Amaranthus blitum	Ter
Cowpeas	Kunde	Vigna unguiculata	Kun
Jute mallow	Mlenda Or Mrenda	Corchorus olitorius	Mur
Black night shade	Mnavu, Managu, Osuga,	Solanum scabrum	Man
Pumpkin leaves	Malenge	Cucurbita moschata	Pum
Slender leaf	Marejea or Mitoo	Crotalaria ochroleuca	Mit
Spider plant	Mgagani or Saget	Cleome gynandra	Sag
Stinging nettles	-	Urticadioica	Sn
Cassava leaves	kisamvu	Manihot esculenta	Cas

## Results

**Figure 1:**
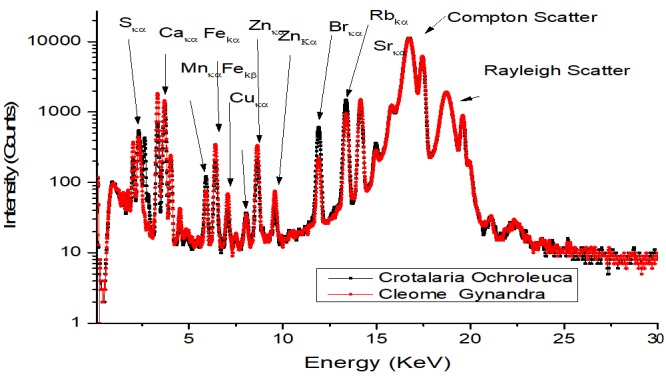
Typical overlaid EDXRFS spectra of Crotalaria ochroleuca and Cleome gynandra vegetable samples

The typical EDXRF spectra of African indigenous leafy vegetable (Crotalaria Ochroleuca and Cleome Gynandra) samples overlaid are shown in Figure 1.

**Method validation using NIST Bowen's Kale as certified reference material:** Table 2 shows the precision of EDXRF used in this study and the Instrument Limit of Detection (ILD) that represents the threshold above which a peak intensity can be distinguished from the background noise at a specified level of confidence [14]. The experimental values are all in good agreement with the certified values due to low calculated relative errors (%) for Bowen kale. This therefore clearly indicate a validated reproducible method for analysis of micronutrient levels in African indigenous leafy vegetables with guaranteed accurate results.

**Table 2:** Experimental and certified values of micronutrients in Bowen’s Kale reference material (Ken/0/003/S.636 F, 1983)

Elements	Experimental values ± deviation (ppm)	Certified values ± deviation (ppm)	Relative error (%)	*ILD (ppm)
Zinc	32.40 ± 1.61	32.29 ± 2.2	0.34	1.38 ± 0.14
Iron	116.60 ± 4.70	119.30 ± 14.80	2.23	5.84 ± 0.50
Magnesium	1597 ± 222	1605 ±181	0.50	297.78 ± 51.04
Calcium	41433 ±723	41060 ± 2220	0.90	59.13 ± 4.14
Potassium	24333 ± 351	24370 ± 1452	0.15	57.75 ± 9.93

* Instrument Limit of Detection represents a threshold above which a peak intensity can be distinguished from the background noise at a specified level of confidence [28]

### Mean (n=12) concentration (mg/g) of micronutrients in African leafy vegetables

**Figure 2:**
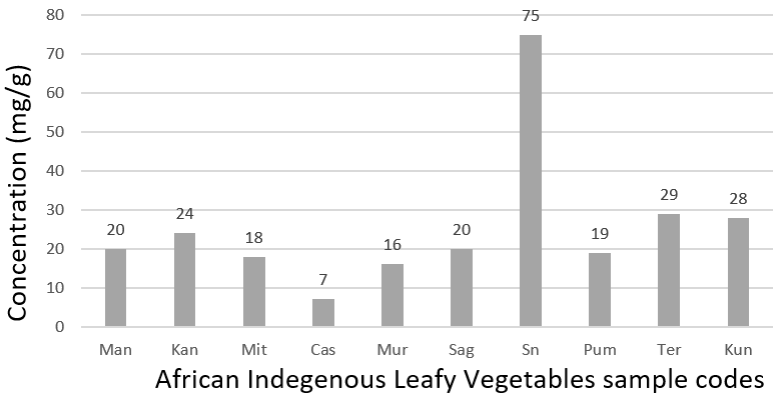
Level of calcium in mg/g in the African indigenous leafy vegetables analysed in the present study

**Calcium:** Figure 2 shows the concentration of Ca in ten African indigenous leafy vegetables. Urticadioica stands out with a level of calcium (75 mg/g) almost 11 times greater than the level of calcium in the Manihot esculenta leaves (7 mg/g) which is the lowest. Amaranthus blitum and Vigna unguiculata have almost the similar amount of calcium, 28 mg/g and 29 mg/g respectively. Same apply to Solanum scabrum, Cleome gynandra and Cucurbita moschata leaves with 20 mg/g and 19 mg/g respectively. Although other related studies showed that calcium requirement may vary from culture to culture for dietary, genetic, lifestyle, and geographical reasons [15], calcium salts provide rigidity to the skeleton and calcium ions play a role in many, if not most, metabolic processes. Calcium deficiency is known to destroy bone, hence contributing to bone resorption. This study clearly demonstrates that people living in Kenya and in the African continent can rely on the AILVs as a source of calcium. Unfortunately, there is very little literature on calcium daily requirement on data derived from African countries. Balk *et al*. reported that countries in Africa and South America mostly have low calcium intake between about 400 and 700 mg/day [16]. Recommended calcium allowances for adults based on North American and Western European data are between 1000 mg/day to 1300mg/day with the highest amount for post menopause females and males beyond 65 years old [15]. A 10 g dry weight of Urticadioica leaves with the highest concentration of calcium (75 mg/g), can provide 75% and 58% of the daily value of calcium based on the North American and western European recommendation.

**Figure 3:**
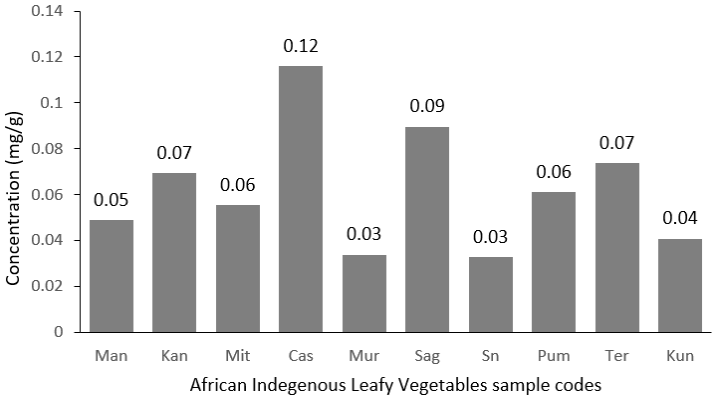
Level of zinc in mg/g in the African indigenous leafy vegetables analysed in the present study

**Zinc:** the level of zinc was assessed in the African indigenous leafy vegetables as shown in Figure 3. Zinc plays a central role in cell division, protein synthesis, growth in infants, children, adolescents, and pregnant women. Prevention of sub-optimal zinc status and zinc deficiency in children by an increased intake and availability of zinc through AILVs consumption could consequently have a significant effect on child health in developing countries. Figure 3 shows a distribution of zinc in the AILVs with Manihot esculenta leaves displaying the highest concentration of 0.1 mg/g. Urticadioica and Corchorus olitorius are at the bottom with an equal zinc amount of 0.03 mg/g. Table 3 present the recommended nutrient intakes to meet the normative storage requirements from diets differing in zinc bio-availability and the corresponding percentage daily value of zinc in 10 g of Manihot esculenta leaves (dried weight). Bio-availability refers to the fraction of intake that can be absorbed into the blood system and used for physiological functions of the body [17]. For zinc, in healthy individuals, it is determined by three factors: the individual's zinc status, the total zinc content of the diet, and the availability of soluble zinc from the diet's food components [18]. Although green leafy vegetables are only modest sources of zinc < 10mg/g [15], they can contribute significantly to the recommended nutrient intake (RNI) for dietary zinc. 10g of Manihot esculenta leaves (dried weight) contains 39% of the daily value of zinc for females aged 19 to 65 and 28% for males in the same age range.

**Table 3:** Recommended nutrient intakes (RNIs) for dietary zinc (mg/day) and corresponding percentage daily value

	^a^Recommended nutrient intakes (RNIs) for dietary zinc (mg/day) to meet the normative storage requirements from diets differing in zinc bio-availability				
Adults	Assumed body weight, kg	^b^High bioavailability	^c^Moderate bioavailability	^d^Low bioavailability	% Daily Value of zinc in 10 g of Manihot esculenta leaves (dried weight)
Females, 19-65 years	55	3.0	4.9	9.8	39%/24%/12%
Males, 19-65 years	65	4.2	7.0	14.0	28%/17%/8%
Females, 65+ years	55	3.0	4.9	9.8	39%/24%/12%
Males, 65+ years	65	4.2	7.0	14.0	28%/17%/8%

a Recommended nutrient intakes (RNIs) for dietary zinc (mg/day) to meet the normative storage requirements from diets differing in zinc bio-availability where extracted from [14]

b Assumed bio-availability of dietary zinc 50 percent.

c Assumed bio-availability of dietary zinc 30 percent.

d Assumed bio-availability of dietary zinc 15 percent [14]

**Figure 4:**
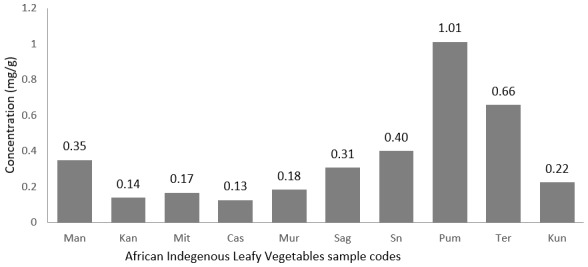
Level of Iron in mg/g in the African indigenous leafy vegetables analysed in the present study

**Iron:** Figure 4 shows the levels of iron in mg/g in the African indigenous leafy vegetables analyzed in the present study. Cucurbita moschata had the highest iron content of 1.01mg/g when Manihot esculenta leaves exhibited the smallest iron content of 0.13mg/g. The recommended nutrient intakes for iron varied with dietary iron bio-availabilities, age and body weight. Table 4 presents the % Daily Value of iron in 10 g of Cucurbita moschata leaves (dried weight). About 10g of Cucurbita moschata leaves (dried weight) provides between 57% to 136% daily value of iron for children age between 7 and 10 years old and can provide up to 41%, 81% and 27% daily value of iron for female aged 18+, lactating females aged 18+ and males aged 18+ respectively. This is very important knowing that worldwide the highest prevalence figures for iron deficiency (being defined as an absence of iron stores) are found in infants, children, teenagers, and women of childbearing age. With an overall 600-700 million people presenting a marked iron deficiency anemia [19] this makes iron deficiency the most frequent nutritional deficiency disorder in the world. Many developed countries have taken actions against iron deficiency through information and access to fortified cereals for infant and children. Cucurbita moschata leaves with its higher content in iron could be considered in developing countries extensive programmes to combat iron deficiency in areas where iron deficiency prevalence is still very high and the severity of anaemia marked [20].

**Table 4:** Recommended nutrient intakes (RNIs) for dietary Iron (mg/day) and corresponding percentage daily value

			^a^Recommended Nutrient Intake (mg/g)			
			% Dietary Iron Bio-availability			
Group	Age	Assumed body weight (kg)	12	10	5	% Daily Value of Iron in 10 g of raw Cucurbita moschata leaves (dried weight)
Children	7-10	28.1	7.4	8.9	17.8	136/113/57
Females	18+	62	24.5	29.4	58.8	41/34/17
Males	18+	75	11.4	13.7	27.4	11/14/27
Lactating Females	18+	62	12.5	15	30	81/67/34

a Recommended nutrient intakes for iron based on varying dietary iron bio-availabilities extracted from[14]

**Figure 5:**
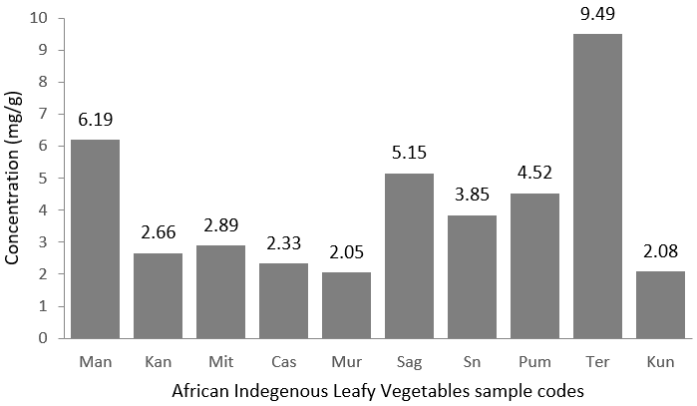
Level of magnesium in mg/g in the African indigenous leafy vegetables analysed in the present study

**Magnesium:** Figure 5 below presents the results of magnesium content found in the leafy vegetables. Amaranthus blitum has the highest concentration of 9.49 mg/g followed by Solanum scabrum and Cleome gynandra with concentrations 6.19 mg/g and 5.15 mg/g respectively. Although dietary deficiency of magnesium of a severity sufficient to provoke pathologic changes is rare [21,22] it is still very important to source African indigenous leafy vegetables that are good source of magnesium and Table 5 presents recommended nutrient intakes for magnesium in milligrams (mg) alongside of the percentage daily Value of Magnesium in 10 g of Amaranthus blitum leaves (dried weight). Amaranthus blitum with more than 20% daily value of Magnesium in 10 g (dried weight) for all adult aged groups is classify as a high magnesium contributor.

**Table 5:** Recommended nutrient intakes (RNIs) for dietary magnesium (mg/day) and corresponding percentage daily value

Recommended nutrient intakes for magnesium in milligrams (mg)			% Daily Value of Magnesium in 10 g of Amaranthus blitum leaves (dried weight)
Adults (years)	Assumed body weight kg	RNI mg/day	
Females 19-65	55	220	43
Females 65	54	190	50
Males 19-65	65	260	37
Males 65+	64	224	42

**Figure 6:**
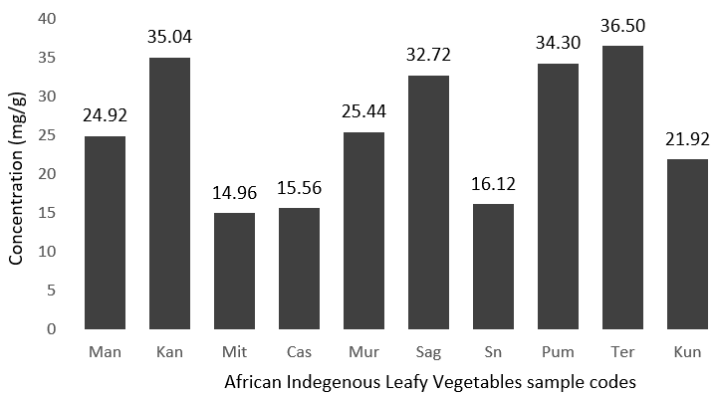
Level of potassium in mg/g in the African indigenous leafy vegetables analysed in the present

**Potassium:** potassium level was assessed in the African indigenous leafy vegetables as shown in Figure 6. Four species, Amaranthus blitum, Brassica carinata, Cleome gynandra and Cucurbita moschata leaves had higher potassium content of 35.60 mg/g, 35.04 mg/g, 34.30 mg/g and 32.72 mg/g respectively. WHO suggests a potassium intake of at least 90 mmol/day (3510 mg/day) for adults. The recommended potassium intake of at least 3510 mg/day should be adjusted downward for children, based on the energy requirements of children relative to those of adults. Based on the 3510 mg daily recommendation, 10 g of Amaranthus blitum, Brassica carinata, Cleome gynandra and Cucurbita moschata leaves (dried weight) will provide 10.4%, 10.0%, 9.8%, and 9.3% daily value for potassium respectively. These four African indigenous leafy vegetables are very good potassium contributors and should be advertised owing to the fact that low potassium intake has been associated with a number of non-communicable diseases (NCDs), including hypertension, cardiovascular disease, chronic kidney stone formation and low bone-mineral density [23]. Low potassium intake may play an important role in the genesis of high blood pressure and cardiovascular diseases [24].

## Discussion

From the results obtained, different types of vegetables have different ability to adopt and accumulate micronutrients. Among the micronutrients assessed in the present study, zinc (Zn) and iron (Fe) are heavy metals and despite their importance as they are necessary for various biochemical and physiological processes in the body [25], they are toxic above certain concentration. The level of Zn in all the leafy vegetables studied was below the permissible limit. Iron content was below the maximum threshold value of 0.70mg/g in all the leafy vegetables except for Cucurbita moschata that exhibited a concentration of 1.01 mg/g. Schönfeldt *et al*. [26] studied the nutrient content of five traditional South African dark green leafy vegetables and reported a high content of iron in analysed samples. They attributed the high iron content to the use of chicken and cattle manure. Jaarsveld *et al*. [27] suggested in their study of nutrient content of eight African leafy vegetables that large variation of iron content in leafy vegetables should in general be interpreted with caution particularly for ground-level growing leaves such as Cucurbita moschata because soil contamination may occur. The concentration of Zn in Amaranthus blitum found in this study is very close to the value found in samples collected from different farms (0.061 mg/g) and markets (0.055 mg/g) in Zanzibar [28] and analysed using EDXRFS. The values of micronutrients obtained for jute mallow (Corchorus olitorius), Pumpkin leaves (Cucurbita moschata), black nightshade (Solanum scabrum) and cowpea (Vigna unguiculata) from this study in comparison to those available in the published literature showed much disagreement with those reported by Jaarsveld et al. Variations in the chemical composition of leafy vegetables, is influenced by the environment, the antropogenic activities and farming practices. Other sources of differences could be attributed to the age of plants at harvest, which affects their genetic composition [27].

## Conclusion

The assessment of the ten African indigenous leafy vegetables confirmed that they are great sources of micronutrients like Calcium, Zinc, Iron, Magnesium and Potassium and can provide for some of them up to 100% of the recommended daily value. There is a need to promote their consumption and marketing through extensive production and knowledge sharing regarding their nutritional importance. This would enhance household nutritional security thereby reducing micronutrient deficiency. Another important finding of this research is that a single vegetable does not hold the highest content of all the micronutrients required for their health maintenance and protective properties. Amaranthus blitum has the highest content of potassium and magnesium. Urtica dioica , Manihot esculenta leaves and Cucurbita moschata leaves haves the highest level of calcium, zinc and iron respectively. It is then important to create awareness about the need for diversification of AILVs in household diets for improved health owing to the fact that consumption of different varieties is influenced by the cultural backgrounds, hence some varieties are only found in certain communities.

### What is known about this topic

- Vital role of micronutrients in the maintenance of good health and enhancement of the immune system for prevention of diseases;
- Plants in general and therefore African indigenous leafy vegetables can be great sources of micronutrients;
- Energy dispersive X-ray fluorescence spectroscopy is a spectro-analytical technique for simultaneous elemental and quantitative analysis in a wide range of sample types based on measurement of characteristic X-ray intensities emitted by the elements in the samples.

### What this study adds

- Energy Dispersive X-Ray Spectroscopy for qualitative and quantitative analysis of selected micronutrients in ten African indigenous leafy vegetables;
- Concentration of Zinc (Zn), Iron (Fe), Magnesium (Mg), Calcium (Ca) and Potassium (K) in ten African indigenous leafy vegetables (AILVs) (Corchorus olitorius, Cucurbita moschata, Amaranthus blitum, Brassica carinata, Cleome gynandra, Solanum scabrum, Crotalaria ochroleuca name the other AILVs here) which are cultivated, marketed and consumed in Kenya and across East and West Africa;
- Daily value of calcium, Zinc, iron, magnesium and potassium provide by 10 g (dried weight) of Urticadioica, Manihot esculenta leaves, Cucurbita moschata, Amaranthus blitum which are the leafy vegetables with the highest content of the respective micronutrient.

## Competing interests

The authors declare no competing interests.
